# Scalable Production of Mechanically Robust Antireflection Film for Omnidirectional Enhanced Flexible Thin Film Solar Cells

**DOI:** 10.1002/advs.201700079

**Published:** 2017-05-05

**Authors:** Min Wang, Pengsha Ma, Min Yin, Linfeng Lu, Yinyue Lin, Xiaoyuan Chen, Wei Jia, Xinmin Cao, Paichun Chang, Dongdong Li

**Affiliations:** ^1^ Shanghai Advanced Research Institute Chinese Academy of Sciences 99 Haike Road Zhangjiang Hi‐Tech Park, Pudong Shanghai 201210 China; ^2^ University of Chinese Academy of Sciences Beijing 100039 China; ^3^ Xunlight (Kunshan) Company Limited Suzhou 215301 China; ^4^ Department of Creative Industry Kainan University No. 1, Kainan Road Luchu Taoyuan County 338 Taiwan

**Keywords:** antireflection films, broadband and omnidirectional, microstructures, roll‐to‐roll imprinting, solar cells

## Abstract

Antireflection (AR) at the interface between the air and incident window material is paramount to boost the performance of photovoltaic devices. 3D nanostructures have attracted tremendous interest to reduce reflection, while the structure is vulnerable to the harsh outdoor environment. Thus the AR film with improved mechanical property is desirable in an industrial application. Herein, a scalable production of flexible AR films is proposed with microsized structures by roll‐to‐roll imprinting process, which possesses hydrophobic property and much improved robustness. The AR films can be potentially used for a wide range of photovoltaic devices whether based on rigid or flexible substrates. As a demonstration, the AR films are integrated with commercial Si‐based triple‐junction thin film solar cells. The AR film works as an effective tool to control the light travel path and utilize the light inward more efficiently by exciting hybrid optical modes, which results in a broadband and omnidirectional enhanced performance.

## 1 Introduction

The efficient light management is of special interest in improving the performance of photovoltaic (PV) devices.^1^, ^2^, ^3^, ^4^ A variety of state‐of‐the‐art light management strategies to increase the optical path length have been developed by random or periodic micro/nanostructures, which could enable the scattering effect, surface plasmonic resonance, and photonic modes.^5^, ^6^, ^7^, ^8^, ^9^, ^10^, ^11^ In order to reduce the reflection of incident light, antireflection (AR) coating has been also widely used on the surface of solar cells^12^, ^13^ and their modules.^14^, ^15^

In the conventional rigid modules covered with glass windows, the interference AR coating is a common approach to reduce the intereface reflection.^16^, ^17^ The typical AR layers are porous SiO_2_ or TiO_2_ films with the effective refractive index between 1.0 (air) and 1.5 (glass) realized by high‐temperature processing.^16^, ^18^ According to the quarter‐wavelength principle of linear optics, an improved transmittance under a specific wavelength (λ) can be achieved with the coating thickness of λ/4.^19^ As a result, multiple coatings^15^, ^20^ with different refractive indices for each layer is needed for broadband AR. Besides the cost issue, the mismatch of the coefficient of thermal expansion among different materials is a technical challenge.^21^, ^22^ Furthermore, under a large incident angle, which is the normality of practical operation, the AR effect would be significantly decreased.^23^, ^24^

Note that the window materials of flexible solar cells are commonly transparent polymers with much lower temperature tolerance^25^, ^26^ ruling out the conventional high‐temperature process. Inspired by the moth‐eye structures, the plastic AR films with 3D subwavelength structures have been proposed.^27^, ^28^, ^29^, ^30^, ^31^, ^32^ The 3D nanostructures provide gradually changing effective refractive index from the top of the nanostructure to the bulk material, which distinctly suppress the reflection omnidirectionally at the air/window interface.^29^, ^33^

Although these nanostructures demonstrated appealing results in improving device efficiency, they suffer from the low mechanical strength that can hardly sustain in the real field environment. In addition, the nanofabrication methods are costly and time consuming with a scale limitation. Unlike the nanostructured textures, microstructures can be produced more efficiently and effectively scatter the incoming light, and in return reduce the surface reflectivity. In this work, we demonstrated the plastic AR films with microstructures fabricated by roll‐to‐roll (R2R) process with large‐area continuous production capability. The microstructures are realized on ethylene‐tetrafluoroethylene (ETFE) films, which are being widely used in flexible PV industry as the polymer front sheet to encapsulate solar cell modules due to its excellent UV resistance, high transmittance, and outstanding tensile strength.^26^ The large area imprinting mold can be readily achieved by computer numeric control (CNC) machining process. Several different structures were produced including stripe‐like triangular prisms, trapezoid prisms, and close‐packed nanopillars structures. Besides the promising omnidirectional AR effect and hydrophobic behavior, the optimal microstructures exhibit improved mechanical durability compared with the nanostructured AR films, which is attractive in the practical industrial application.

## 2 Results and Discussion

**Figure 1:**
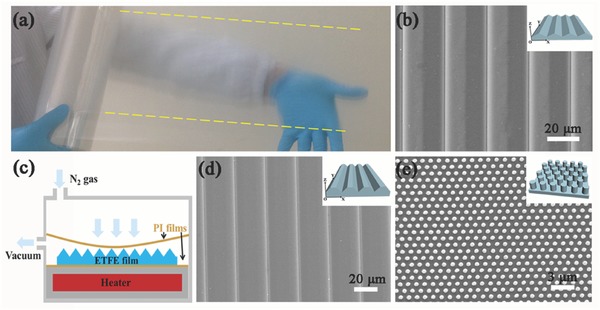
a) The photograph of ETFE film with triangular prisms microstructure. b) SEM image of ETFE film with triangular prisms, c) A schematic illustration of a homemade vessel with a heater, SEM images of ETFE films with d) trapezoid prisms and e) nanopillar arrays structure. The insets in (b), (d), and (e) are their corresponding 3D schematics.

The flat ETFE films with a thickness of 75 µm serve as the starting material. **Figure** **1**a shows the photograph of an ETFE film prototype with a triangular prism texture imprinted. The textured area is 20 cm wide showing by the yellow dash lines that can be further widened by modifying the R2R machine. The film shows a hazy look surface resulted from its triangular structure refracting the light. Figure 1b represents the scanning electron microscope (SEM) image of the film surface showing the line‐space triangular prism structure with the pitch and height of 25 and 5 µm, respectively. The production of stripe‐like structures is much easier than the 3D matrix in view of both the roller molds fabrication during CNC machining process and the demolding effect in the imprinting process. In order to fabricate the trapezoid prism structure, the triangular microstructured film was subsequently hot pressed under 0.3 MPa and 160 °C for 15 min in a homemade double‐chamber setup as illustrated in Figure 1c. One can find a morphology evolution from triangular prism to trapezoid structure with the pitch and height of 25 and 2 µm, respectively (Figure 1d). The trapezoid prism structure with a lower aspect ratio is beneficial to obtain better mechanical durability. Moreover, the hot pressing process is compatible with the lamination process that is widely used for the encapsulation of solar panels.^34^, ^35^ The insets in Figure 1b,d show corresponding 3D schematics, which define line‐space prisms parallel to the *y*‐axis. For the subsequent comparison, the nanopillar structure was also transferred from the nanostructured nickel mold through planar hot embossing process with the pitch size, height, and diameter of 1 µm, 500 and 500 nm, respectively (Figure 1e).

**Figure 2:**
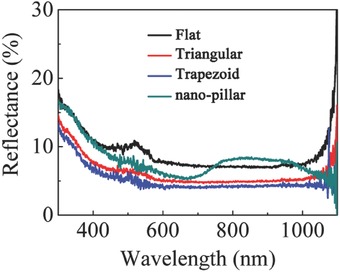
The reflection spectra of ETFE films with different surface morphologies.

**Figure** **2** shows the reflectance spectra of the above ETFE films along with a flat reference, in which the triangular and trapezoid structures exhibit better AR performance in the range of 300–1100 nm with the reflectance of below 4.9% at 600 nm. It is worth noting that the nanopillar structure has a relatively high reflectance in the range of 750–950 nm that could be attributed to the interference of the reflected light on the nanopillar arrays.^36^

**Figure 3:**
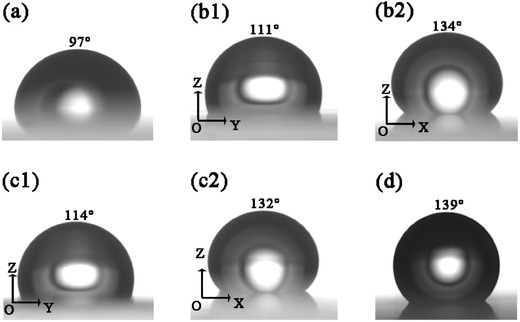
The water CAs of ETFE films with a) flat, b1,b2) triangular prisms, c1,c2) trapezoid prisms, and d) nanopillar structures. The (b1) and (c1) are viewed perpendicular to *yoz* planes, while (b2) and (c2) are viewed perpendicular to *xoz* planes.

The flat film shows hydrophobic property with a water contact angle (CA) of 97° (see **Figure** **3**a) due to the large bond energies of C—F and long perfluoroalkyl side chains of ETFE.^37^ AR films with micro/nanostructures would improve the hydrophobic characteristics with larger CAs compared with the flat one. Due to the anisotropic patterns as illustrated in Figure 1b,d, triangular and trapezoid prisms would have different CAs along different axial directions. Figure 3b,c shows higher CAs for both triangular (CA = 134°) and trapezoid (CA = 132°) prism structure viewed perpendicular to *xoz* planes than that to *yoz* planes and close to nanopillar structure (CA = 139°) (see Figure 3d). The improved hydrophobicity will facilitate the self‐cleaning effect, which decreases the solar panels cleaning cost in practical operation.

**Figure 4:**
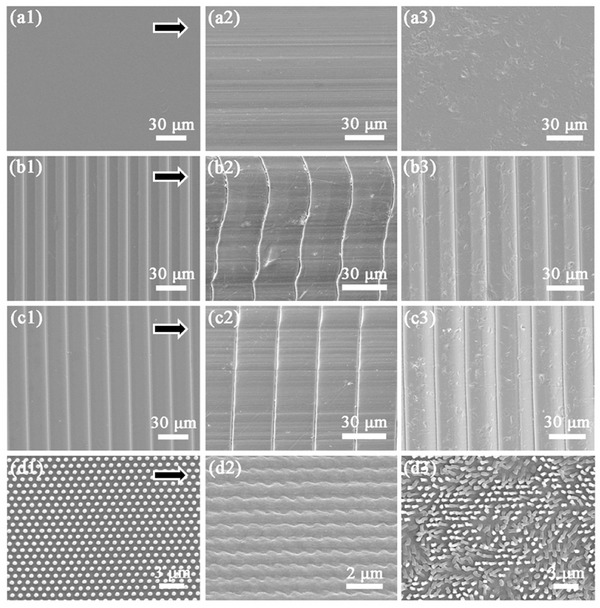
SEM images of ETFE films with a1–a3) flat, b1–b3) triangular prisms, c1–c3) trapezoid prisms, and d1–d3) nanopillar arrays structure a1–d1) before and a2–d2) after pencil scratch, and a3–d3) after sand blasting tests. The arrows in (a1)–(d1) represent the scratch direction of the pencil.

The mechanical durability is crucial to the AR films of solar panels because of the harsh outdoor environment. Due to the low elastic modulus of ETFE (1.2 GPa at 25 °C),^38^ mechanical durability would be a fatal issue especially with textured surface.^38^, ^39^ Pencil scratch tests on four different structures were carried out with the tester hardness of 6B, and the weight of 500 g. The pencil track is in the horizontal direction during all tests, as illustrated by the arrows in **Figure** **4**a1–d1. Obvious pencil tracks were found in all four samples with smeared surface structures as shown in Figure 4a2–d2. The AR film with trapezoid prisms shows the best scratch resistance with a little distortion while nanopillar structure shows the worst resistance performance where all the pillars were squashed and adhered.

The sand resistant characterizations were also performed in view of the dust condition in the outdoor environment that could significantly affect the solar panel performance. The sand blasting tests were conducted with the sand particles size below 350 µm at a speed of 1.4 m s^−1^. The SEM images (Figure 4a3–d3) of four different structures after sand tests indicate that the microstructures (triangular and trapezoid prisms) possess better sand resistance compared to nanopillar structures, where the nanopillars collapsed after the sand test. Moreover, a bending test was also carried out to evaluate the stability of micro/nanostructures of AR films. The bending angle (0°–180°) was automatically controlled using a customized setup (Figure S1, Supporting Information). The micro/nanostructures were intact after 10 000 cycles of bending (Figure S2, Supporting Information), indicating outstanding flexibility and stability. According to the above test results, the AR film with trapezoid structure shows better performance and is chosen to apply on the solar cells for further evaluations.

**Figure 5:**
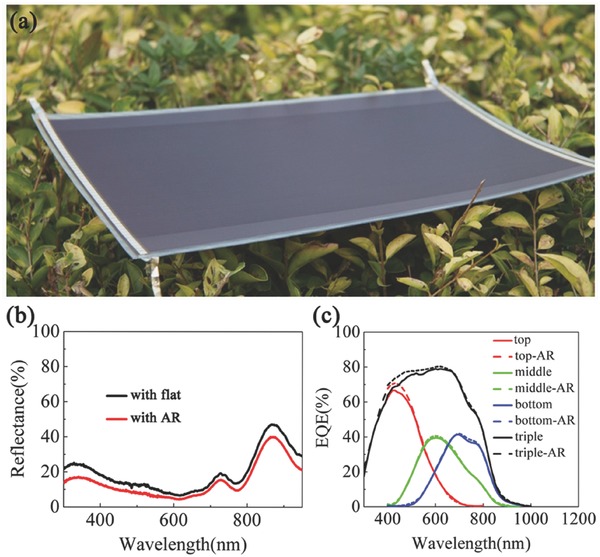
a) A photograph of a three‐junction thin‐film solar cell. b) Reflectance measurements of the devices covered with flat and trapezoid AR films. c) EQE measurements of the devices covered with flat and trapezoid AR films. The three subcells are denoted as “top,” “middle,” and “bottom,” while “triple” represents the overall quantum efficiency.

As a demonstration, the trapezoid AR films were integrated with commercial Si‐based triple‐junction thin film solar cells (Xunlight (Kunshan) Co., Ltd).^40^ The encapsulation is realized by laminating (see details in the Experimental Section) the triangular prism ETFE film and thin film solar cell with the help of ethylene vinyl acetate (EVA) as an adhesive layer. The surface profile is coincident with that obtains from direct hot pressing process under the same pressure and temperature. **Figure** **5**a represents an ETFE encapsulated thin‐film solar cell (24 × 35.5 cm^2^) partially covered with the trapezoid AR structure. At the area covered by the trapezoid prisms, one can clearly see the significant AR effect improvement. The normal incident reflectance spectra are measured on the devices covered with flat and trapezoid AR films as shown in Figure 5b. It is seen that the optical reflection is substantially suppressed over the whole wavelength region after introducing the AR film. Figure 5c shows the external quantum efficiency (EQE) results of the device with flat and trapezoid AR films at a normal incident angle. The calculated *J*_SC_ of three subcells by integrating Air Mass (AM) 1.5G spectrum^41^ is 6.94 (top cell), 6.68 (middle cell), and 6.47 (bottom cell) mA cm^–2^ for the device with flat film, while it is 7.2, 6.80, and 6.53 mA cm^–2^ for the device with AR film. This indicates that the AR film benefits a higher improvement for the top cell, which utilizes comparative short wavelength.

**Figure 6:**
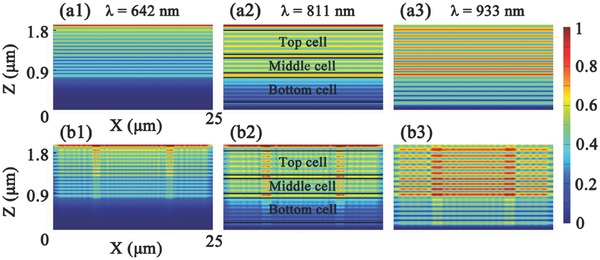
The cross‐sectional electric field intensity distributions in the devices covered with a1–a3) flat and b1–b3) trapezoid AR films at different wavelengths: a1,b1) 642, a2,b2) 811, and a3,b3) 933 nm. The black lines in (a2) and (b2) represent the subcells of the triple‐junction solar cell.

The optical light field behaviors are verified by electromagnetic simulation via finite‐difference‐time‐domain (FDTD) method. The simulation for the structures of solar cells laminated with flat and AR film were depicted in Figure S3 (Supporting Information), where the thickness of flat film and the flat part in the trapezoid AR film are reduced in order to save computational resource without losing accuracy. **Figure** **6** shows the cross‐sectional electric field (|*E*|) distributions of the triple‐junction solar cells at the wavelengths of 642, 811, and 933 nm, respectively, where the guidelines in Figure 6a2,b2 indicate the subcells of the triple‐junction device.

A series of finger patterns along the *z*‐axis (i.e., vertical direction) can be found in the flat device (Figure 6a1–a3), originated from the interference between the incident and reflected light. The interference effects are also called cavity modes, which are formed inside the cells due to the different refractive index of each cell.^30^ As the incident wavelength becomes longer, the cavity modes will penetrate into the bottom cell (Figure 6a2,a3). With the presence of AR film, the cavity modes also emerge into the cells along the vertical direction as shown in Figure 6b1–b3, but the interference fringes are spot shaped and distributed evenly along the *x*‐axis (i.e., horizontal direction) instead of straight across the cells. The constructive modes formed along the horizontal direction were defined as Bloch modes resulted from the periodical structure of the AR film (i.e., the lattice scattering effect),^42^ which benefits light trapping in cells. Conclusively, the absorption enhancement in the device with AR film attributes to the hybrid modes combining the cavity mode and the Bloch mode.

**Figure 7:**
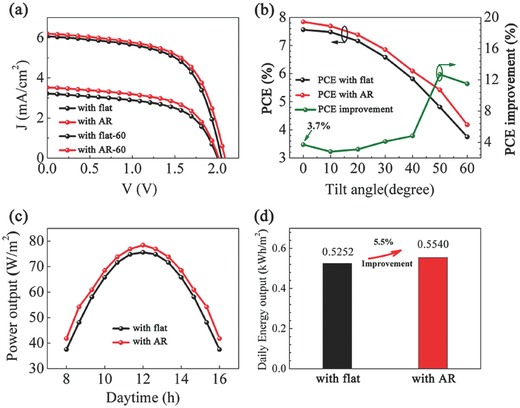
a) The *J–V* measurements of the devices encapsulated with flat and trapezoid AR films under 0° and 60° incident angles. b) The PCE values of the devices encapsulated with flat and trapezoid AR films obtained at different incident angles, together with the angular‐dependent improvement of PCE. c) Power output of the devices encapsulated with flat and trapezoid AR films against daytime. d) Daily energy output of the devices encapsulated with flat and trapezoid AR films. All the incident angles are varied in *yoz* plane.

It is important to understand that the angle of solar irradiation changes over the time during a day in practical operation. Therefore, omnidirectional light trapping ability of AR film is critical. Due to the anisotropic characteristic of trapezoid structure, the incident light angle dependent performances are characterized in both *yoz* and *xoz* planes. As the power conversion efficiency (PCE) and the internal resistance of solar cells were related to cell size and conductivity.^43^ A large size solar cell (such as Figure 5a) would experience a PCE decrease due to the possible defects. In this work, the solar cells with a smaller size (area: 4.4 × 5 cm^2^) are used in the performance evaluations. The small area devices are cut from the same large size solar cell in order to minimize the performance variation. **Figure** **7**a illustrates the current density–voltage (*J*–*V*) curves of the devices recorded at 0° (normal incidence) and 60° of incident light (in *yoz* plane) under AM 1.5G solar simulator irradiation, where the current densities are calculated using the device active area, regardless of the change of projection area under different incident angles. A series of *J*–*V* measurements were further performed at different incident angles varying from 0° to 60° with a 10° interval in *yoz* plane.^30^ The incident angle dependent devices performances, together with the relative improvement, are summarized in Figure 7b and Table S1 (Supporting Information). It is shown that the incorporation of AR films leads to a 3.7% improvement of PCE at a normal incident angle, benefiting from the intensified electric field intensity as depicted in Figure 6. As the incident angle increased, all the PCE values are reduced, while the significance of AR film is positively correlated with the increased incident angle. Under an incident angle of 60° in *yoz* plane, one can find an 11.5% enhancement of PCE compared with its flat counterpart.

More intriguingly, we found an angle dependence of the AR film (Table S1, Supporting Information) when applied in *xoz* plane due to the anisotropic trapezoid geometry. Under 60° incident angle in *xoz* plane, the PCE value (3.70%) with AR film is slightly lower than that with flat window layer, which could be attributed to a higher reflection at a specific angle. As a result, it is preferable to have the incident irradiation move in *yoz* plane. This behavior is adoptable for practical application because one can have the motion trajectory of the sun approximately fall into the vertical plane with respect to the solar panel.

Figure 7c shows the power output of the devices encapsulated with flat and trapezoid AR films, where 0°–60° (in *yoz* plane) is corresponding to 12 pm to 4 h away from 12 o'clock (from 8:00 am to 16:00 pm).^44^ The device with AR film demonstrates an all‐day improvement of electrical power output. Figure 7d shows daily energy output difference of the two devices that was calculated by integrating the power output of the device from 0° to 60° of incident angles (from daytime 8 to 16 o'clock)^45^

(1) $$E_{\mathrm{Daily}}\;=\;\sum_{8}^{16}P_{\mathrm{output}}\;\times \;0.667\;h$$

where *E*_Daily_ is the daily energy output, *P*_output_ is the power output at designated daytime, and 0.667 h is the time interval corresponding to 10° incident angle change. The daily energy output of 0.5540 kW h m^−2^ is achieved with the presence of AR film, which outperforms the planar counterpart by 5.5%. Although the PCE improvement with AR film is not as high as that with previous nanostructured 3D AR films,^45^ AR film with microscale trapezoid prisms shows the prospective scalable application with the advantages of lower production cost and higher mechanical strength.

## 3 Conclusion

In summary, we have demonstrated large‐area flexible AR films with microprism structures by a versatile R2R imprinting process. Large‐area microscale trapezoid structure can be easily transferred through roller molding and hot pressing fabrication resulting in reduced cost and prolonged the service life of the mold. The AR films delivered broadband suppression of light reflectance together with improved hydrophobic property that can be easily integrated with flexible and rigid solar cells. The solar cell with the presence of AR film demonstrates a 5.5% energy generation enhancement over the device with flat encapsulation film. The AR film shows attractive application prospects with much enhanced mechanical strength and can be easily utilized on many commercial solar panels.

## 4 Experimental Section

*Fabrication of AR Films with R2R Process*: The AR films are fabricated by an R2R imprinting equipment, as shown in Figure S6a (Supporting Information). A copper‐coated steel roller mold (Figure S6b, Supporting Information) was made by CNC lathe technology, whose surface had triangular prisms microstructure (pitch = 25 µm and height = 5 µm). The roller mold was used as the driver roller, where a heater was inserted into its core. The microstructure was transferred from the rollers to ETFE films at 120 °C with a pressure of 0.5 MPa. After cooling down, the polymer with microstructure demolds from the roller. In order to realize the trapezoid prism structure, a hot pressing process was subsequently carried out in a homemade vessel with a heater (Figure 1c). The triangular prism film served as the starting material, which is sandwiched in between two flat polyimide (PI) films. The upper PI film also served as a separator to isolate the chamber into two parts. The hot pressing was conducted where the elevated temperature (160 °C) was achieved by a well‐controlled heater. The applied pressure (0.3 MPa) was realized by vacuum pumping the bottom chamber and injection of high‐pressure nitrogen gas into the upper chamber.

*Encapsulation of Thin Film Solar Cells with AR Films*: The AR film with trapezoid prisms was obtained by laminating the triangular prism on the triple‐junction thin film solar cell. Briefly, AR film with triangular prisms, EVA sheet (500 µm thickness), and triple‐junction thin film solar cell (provided by Xunlight (Kunshan) Co. Ltd) were stacked together in order. They were equipped with a double‐chamber setup as illustrated in Figure 1c. The upper and down chamber were separated by a PI film. Both chambers were first vacuumed (≈0.1 MPa) by a mechanical pump for 10 min to eliminate the possible air among these layers. Then the vessel was heated to 120 °C and kept for 5 min. After that, the upper chamber was filled with N_2_ gas with the pressure of 0.2 MPa. The overall applied pressure was 0.3 MPa, plus the pressure of 0.1 MPa from down chamber. The temperature increased from 120 to 160 °C and kept for 15 min. Finally, the solar cell encapsulated with ETFE film was cooled to room temperature.

*Numerical Simulation*: FDTD method was used to investigate the absorption properties of the devices with or without AR film. The thickness of flat film and the flat part in the trapezoid AR film were reduced in order to save computational resource. A plane wave light source with wavelengths ranging from 300 to 1000 nm was utilized. The unit cell of the compound device was set as the simulation region utilizing Bloch boundaries in the *x*‐ and *y*‐axis a Perfectly Matched Layer (PML) boundaries in the *z*‐axis at a normal incident angle. The optical data used in this simulation were obtained from Palik's Handbook of Optical Constants^46^ and online database.^47^

*Characterization*: The SEM images were taken using cold field emission scanning electronic microscopy (S‐4800, Hitachi) at 3.0 kV. The reflectance spectra were measured by a fiber‐optic spectrometer (Maya 2000 Pro, Ocean Optics). The contact angle of the polymer film was measured by a Kruss Kontaktwinkle DSA100 setup. The *J–V* characteristic of solar cells was carried out under 25 °C using an Xe lamp solar simulator (Newport, 94063A‐1000, 100 mW cm^−2^) coupled with an air mass 1.5 global (AM 1.5G) filter. The external QE measurements of the solar cell were gained by QEX10 (PV Measurements, Inc.).

## Conflict of Interest

The authors declare no conflict of interest.

## Supporting information

Supplementary
