# Supplementary material for: Scalable Production of Mechanically Robust Antireflection Film for Omnidirectional Enhanced Flexible Thin Film Solar Cells
Source: Adv Sci (Weinh). 2017 May 5;4(9):1700079. doi: 10.1002/advs.201700079 (PMC5604369; doi:10.1002/advs.201700079)
Supplement: Supplementary file 1 — Supplementary [file ADVS-4-na-s001.pdf]

Copyright WILEY-VCH Verlag GmbH & Co. KGaA, 69469 Weinheim, Germany, 2017.

ADVANCED SCIENCE
Open Access

Supporting Information

for *Adv. Sci.*, DOI: 10.1002/advs.201700079

Scalable Production of Mechanically Robust Antireflection Film for Omnidirectional Enhanced Flexible Thin Film Solar Cells

Min Wang, Pengsha Ma, Min Yin, Linfeng Lu, Yinyue Lin, Xiaoyuan Chen, Wei Jia, Xinmin Cao, Paichun Chang, and Dongdong Li**

Copyright WILEY-VCH Verlag GmbH & Co. KGaA, 69469 Weinheim, Germany, 2016.

Supporting Information

Scalable Production of Mechanically Robust Antireflection Film for Omnidirectional Enhanced Flexible Thin Film Solar Cells

Min Wang, Pengsha Ma, Min Yin, Linfeng Lu, Yinyue Lin, Xiaoyuan Chen, Wei Jia, Xinmin Cao, Paichun Chang, Dongdong Li **

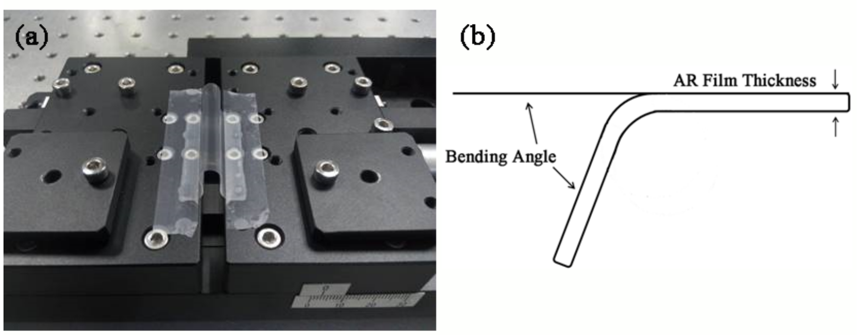

Figure S1. (a) The photograph of a bended AR film with trapezoid prism mounted on the measurement setup. (b) An illustration schematic of defining bending angle.

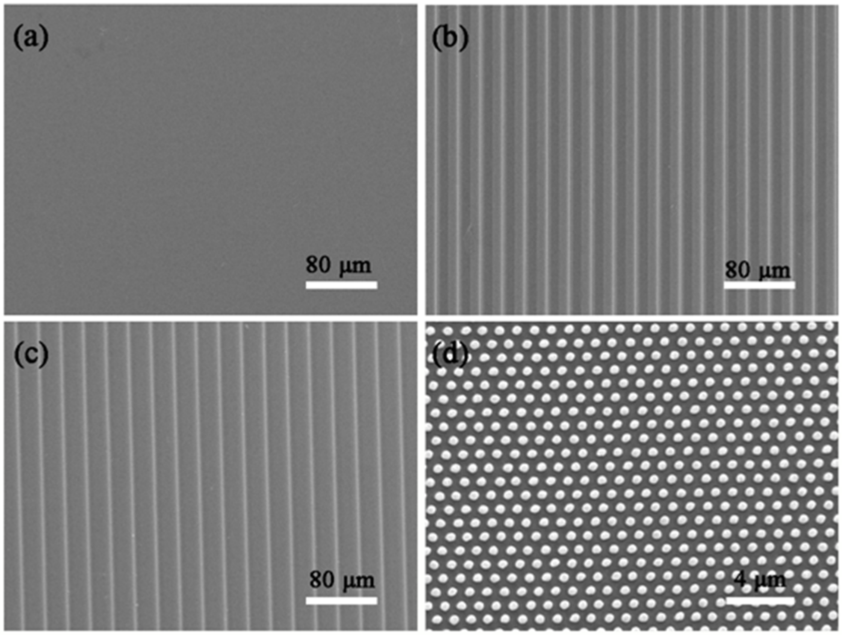

Figure S2. SEM images of ETFE films after bending test (a) flat, (b) triangular prisms, (c) trapezoid prisms, (d) nano-pillar arrays structure.

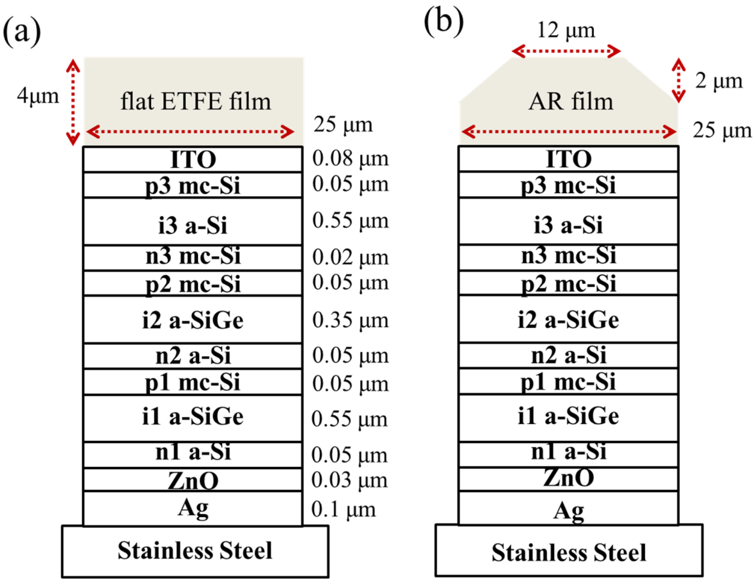

Figure S3. Structure schematics of the triple-junction thin film solar cells covered with (a) flat and (b) trapezoid AR films.

Table S1. The performances of devices encapsulated with flat and trapezoid AR films measured at different incident angles. Due to the anisotropic characteristic of trapezoid structure, the *J*-*V* measurements were carried out in both *yoz* and *xoz* (Figure S4) planes.

Parameters	Device	Angle (degree)						
		0	10	20	30	40	50	60
J_{SC} (mA cm^{-2})	Without AR	6.08	6.0	5.78	5.35	4.77	4.02	3.22
	With AR (*yoz*)	6.21	6.12	5.92	5.51	4.95	4.44	3.53
	With AR (*xoz*)	6.21	6.14	5.89	5.44	4.82	4.12	3.18
V_{OC} (V)	Without AR	2.05	2.05	2.05	2.04	2.04	2.02	2.00
	With AR (*yoz*)	2.09	2.08	2.07	2.06	2.05	2.04	2.01
	With AR (*xoz*)	2.09	2.07	2.07	2.06	2.05	2.03	2.00
FF	Without AR	0.61	0.61	0.61	0.60	0.60	0.59	0.58
	With AR (*yoz*)	0.61	0.61	0.60	0.60	0.60	0.60	0.59
	With AR (*xoz*)	0.61	0.60	0.60	0.60	0.60	0.59	0.58
PCE (%)	Without AR	7.56	7.48	7.16	6.58	5.81	4.81	3.75
	With AR (*yoz*)	7.84	7.69	7.38	6.85	6.09	5.42	4.18
	With AR (*xoz*)	7.84	7.67	7.33	6.73	5.91	4.95	3.70

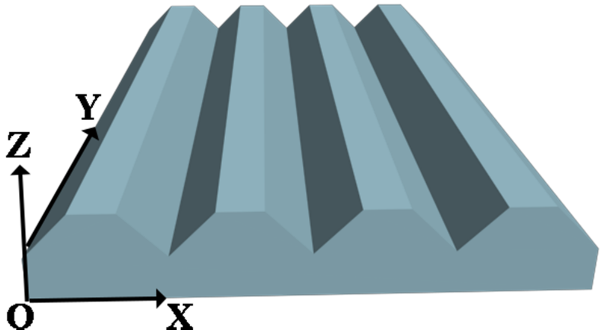

Figure S4. Three dimensional schematic of antireflection film (AR) with trapezoid prism.

A sand self-cleaning experiment was carried out to verify the function of the AR film, and was shown in Figure S5a. Sand particles can be easily removed from the 20°- tilt device surface. It demonstrates that self-cleaning function can be achieved on the solar panel with AR film. The performance of device with AR film was not

influenced by sand test, which varied from 7.98 to 7.92%, as demonstrated in Figure S5b. This further proves of AR film with robust sand resistant ability.

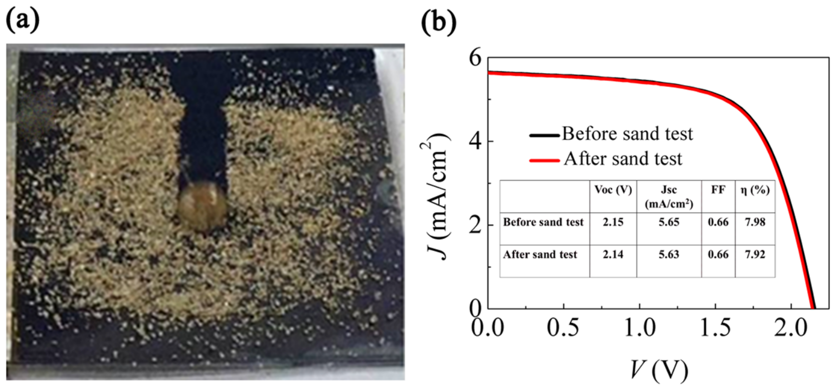

Figure S5. (a) Self-cleaning test of triple-junction thin film solar cell. (b) *J*-*V* measurements of the device with AR film before and after sand test.

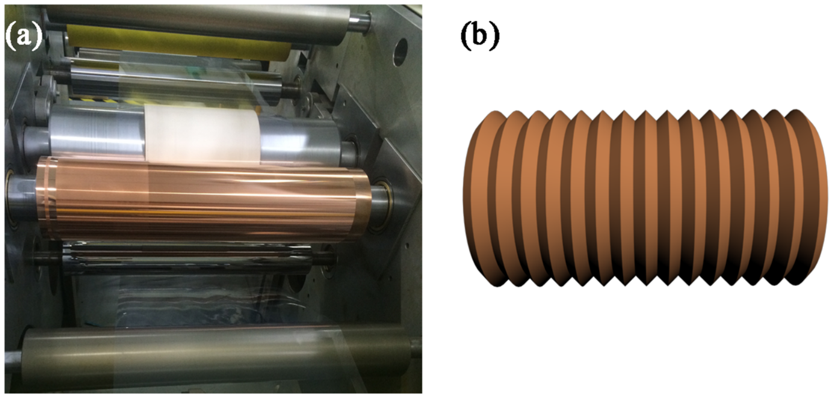

Figure S6. (a) Photograph of R2R imprinting equipment. (b) A schematic illustration of an imprinting roller mold.
